# Long‐term neuroplasticity in language networks after anterior temporal lobe resection

**DOI:** 10.1111/epi.18147

**Published:** 2024-11-06

**Authors:** Maria Sablik, Marine N. Fleury, Lawrence P. Binding, David P. Carey, Giovanni d'Avossa, Sallie Baxendale, Gavin P. Winston, John S. Duncan, Meneka K. Sidhu

**Affiliations:** ^1^ Department of Clinical and Experimental Epilepsy UCL Queen Square Institute of Neurology London UK; ^2^ MRI Unit Chalfont Centre for Epilepsy Chalfont St. Peter UK; ^3^ College of Medicine and Health, Cognitive Neuroscience Institute Bangor University Bangor UK; ^4^ Department of Computer Science UCL Centre for Medical Image Computing London UK; ^5^ Division of Neurology, Department of Medicine Queen's University Kingston Ontario Canada

**Keywords:** ATLR, fMRI, hippocampal sclerosis, longitudinal, plasticity, refractory epilepsy, reorganization, TLE

## Abstract

**Objective:**

Anterior temporal lobe resection (ATLR) is an effective treatment for drug‐resistant temporal lobe epilepsy (TLE), although language deficits may occur after both left and right ATLR. Functional reorganization of the language network has been observed in the ipsilateral and contralateral hemispheres within 12 months after ATLR, but little is known of longer‐term plasticity effects. Our aim was to examine the plasticity of language functions up to a decade after ATLR, in relation to cognitive profiles.

**Methods:**

We examined 24 TLE patients (12 left [LTLE]) and 10 controls across four time points: pre‐surgery, 4 months, 12 months, and ~9 years post‐ATLR. Participants underwent standard neuropsychological assessments (naming, phonemic, and categorical fluency tests) and a verbal fluency functional magnetic resonance imaging (fMRI) task. Using a flexible factorial design, we analyzed longitudinal fMRI activations from 12 months to ~9 years post‐ATLR, relative to controls, with separate analyses for people with hippocampal sclerosis (HS). Change in cognitive profiles was correlated with the long‐term change in fMRI activations to determine the “efficiency” of reorganized networks.

**Results:**

LTLE patients had increased long‐term engagement of the left extra‐temporal and contralateral temporal regions, with better language performance linked to bilateral activation. Those with HS exhibited more widespread bilateral activations. RTLE patients showed plasticity in the left extra‐temporal regions, with better language outcomes associated with these areas. Both groups of patients achieved cognitive stability over 9 years, with more than 50% of LTLE patients improving. Older age, longer epilepsy duration, and lower pre‐operative cognitive reserve negatively affected long‐term language performance.

**Significance:**

Neuroplasticity continues for up to ~9 years post‐epilepsy surgery in LTLE and RTLE, with effective language recovery linked to bilateral engagement of temporal and extra‐temporal regions. This adaptive reorganization is associated with improved cognitive outcomes, challenging the traditional view of localized surgery effects. These findings emphasize the need for early intervention, tailored pre‐operative counseling, and the potential for continued cognitive gains with extended post‐ATLR rehabilitation.

**Key points**

- Left temporal lobe epilepsy (LTLE) patients show long‐term increased engagement in the left extra‐temporal and contralateral temporal regions, with better language linked to bilateral recruitment, suggesting that compensatory neuroplasticity is a mechanism supporting long‐term cognitive recovery.
- In people with right TLE (RTLE), there is increased activation in the left extra‐temporal regions in the long‐term correlates with enhanced language function.
- TLE patients with left hippocampal sclerosis with TLE with left hippocampal sclerosis (vs without) show more widespread bilateral temporal and extra‐temporal activations, including the remnant left posterior hippocampus. RTLE patients show more left extra‐temporal and temporal activation.
- Over ~9 years post‐surgery, the LTLE and RTLE groups maintained cognitive stability. More than 50% of TLE patients showed language improvements, indicating sustained neuroplasticity and potential for continued gains with prolonged anterior temporal lobe resection (ATLR) rehabilitation.
- Lower baseline cognitive function, older age, and longer epilepsy duration—especially in RTLE patients—are linked to greater postoperative decline, highlighting the importance of pre‐surgical cognitive reserve and early surgical intervention.

## 1 INTRODUCTION

Temporal lobe epilepsy (TLE) is the most common drug‐resistant focal epilepsy.^1^ It is associated with widespread structural and functional network dysfunction resulting in cognitive difficulties beyond the epileptogenic temporal lobe. Widespread language function reorganization has been described in people with medically refractory TLE.^2^, ^3^ Epilepsy in the dominant hemisphere is associated with a higher risk of language dysfunction than epilepsy in the non‐dominant hemisphere, particularly the ability to name and generate words.^4^

Functional magnetic resonance imaging (fMRI) is a valid, non‐invasive method for assessing language laterality^5^ pre‐operatively. Verbal fluency together with verb generation tasks are commonly used clinical language paradigms as they reliably activate the left inferior frontal gyrus (IFG) and temporal language areas,^6^ and are highly correlated with contralateral posterior cerebellar regions.^7^, ^8^, ^9^ In TLE, language reorganization has been previously documented in atypical regions, with evidence of reorganization occuring both intrahemis and interhemispherically.^10^ People with left TLE (LTLE) have a higher likelihood of atypical language organization and dynamic reorganization, than in right TLE (RTLE).^11^, ^12^

Anterior temporal lobe resection (ATLR) is an effective treatment for refractory TLE, with up to 80% chance of seizure remission at 12 month of follow‐up^13^; however, there is up to a 50% risk of language decline following dominant ATLR.^14^, ^15^ Decline in language function, specifically in naming, has also been described after non‐dominant resections.^11^

Post‐operative intrahemispheric and interhemispheric plasticity has been shown up to 6 months after ATLR with varied results.^16^, ^17^, ^18^ Bonelli et al.^11^ showed that in LTLE, there is early–4 month–post‐operative language plasticity involving the contralateral (right) IFG. Postoperatively, in LTLE with naming decline, better naming was associated with increased activation in the right middle frontal gyrus. In patients without decline, improved naming correlated with greater activation in the left posterior hippocampus (HC). In RTLE, better naming ability was associated with bilateral frontal involvement, and recruitment of the left HC. Long‐term postoperative functional reorganization has been studied in memory research. Recently, Fleury et al.^19^ demonstrated that long‐term cognitive support following ATLR is mediated by functional network reorganizations, with successful verbal and visual memory encoding at 10 years post‐surgery involving extensive activation of the bilateral temporal and extratemporal regions. The literature on functional imaging of long‐term reorganization in stroke and aphasia is more extensive than that pertaining to TLE. Despite the distinct etiologies of stroke and TLE, including those resulting from ATLR surgery, the neural damage and subsequent language reorganization exhibit notable parallels, suggesting that insights from the stroke literature could be beneficial in understanding TLE outcomes. A recent meta‐analysis by Wilson and Schneck^20^ indicated that longitudinal studies post‐stroke provide some evidence supporting the return of language function to the left‐dominant hemisphere over the long term post‐stroke.^21^

Although language network plasticity has been studied up to 6 months post‐ATLR,^11^, ^17^, ^18^, ^22^, ^23^ reports of post‐operative language outcome in the longer term are varied.^24^ Some authors report progressive cognitive decline continuing 13 years post‐surgery,^14^ whereas others report cognitive stability 10 years post‐operatively.^25^ The main factor associated with worse long‐term language function is poor postoperative seizure outcome.^26^ For memory function, contributing factors include lower preoperative neuropsychological scores, reduced intelligence levels, and longer epilepsy duration.^19^

We studied long‐term longitudinal plasticity (median 9 years) in the language networks after left and right ATLR, and hypothesize the following:

(1) There will be ongoing plasticity in both the left and right TLE groups in ipsilateral and contralateral temporal and frontal regions in the long term after ATLR.^19^
(2) Previous cognitive studies^27^ have shown increased engagement of the posterior HC in memory tasks shortly after surgery; however, in the long term, contralateral neocortical regions became involved in these tasks. We hypothesize that may be true for language network. Moreover, there might be an increase in the left extra‐temporal areas as shown in long‐term post‐stroke studies.^21^
(3) Temporary compensation observed in the initial phases of post‐ATLR recovery,^11^ which involves the non‐lesioned (contralateral) hemisphere, may continue to be supportive in the long‐term.^19^
(4) Preoperative cognitive reserve, longer epilepsy duration, and older age may negatively affect long‐term language outcome.^19^

## 2 MATERIALS AND METHODS

We investigated long‐term postoperative changes occurring 7–10 years (median follow‐up 9 years) after ATLR in TLE compared to changes in controls examined at equivalent intervals.

### 2.1 Participants

We studied 24 people with drug‐refractory TLE (12 left) and 10 controls (CTRL) at four time‐points: (1) pre‐surgery and (2) 4 months after, (3) 12 months after, and (4) 7–10 years after ATLR (median = ~9 years). Patients underwent presurgical assessment at the National Hospital for Neurology and Neurosurgery, London, UK, between 2008 and 2013. The control group was matched in terms of age and gender, as the patients had no previous neurological or mental health conditions. People who were not fluent in English, could not have an MRI, or had an intelligent quotient (IQ) of <70 were not included. All three groups were similar in terms of their demographic characteristics. The International League Against Epilepsy (ILAE) classification of postoperative seizure outcome following epilepsy surgery was used.^28^ There was no difference in the median number of anti‐seizure medications and seizure burden between RTLE and LTLE groups.

Demographic and clinical data are detailed in Table 1.

**TABLE 1:** TLE patients and the control group.

Group	Median (IQR) age, years	Age range, years min–max	Sex, M/F	Mean age at onset (SD), years	Mean epilepsy duration (SD), years	Pathology (*n*)				IQ (SD)	McKenna graded naming *z*‐score at ~9 years post‐op (group mean)	Phonemic fluency *z*‐score at ~9 years post‐op (group mean)	Categorical fluency *z*‐score at ~9 years post‐op (group mean)	No. of patients in class I: 9 years follow up after surgery^a^
						Hippocampal sclerosis	Hippocampal sclerosis + ischemic damage or focal dysplasia	Glial‐neuronal tumors (gangliogliomas, DNET)	Other: e.g., gliosis/cavernoma					
LTLE	37.5 (13.75)	19–50	7/5	19 (12.8)	15.8 (12.2)	9	1	1	1	95.0 (9.53)	−2.01	0.29	−0.48	10/12 (83%)
RTLE	39.5 (17.5)	17–56	4/8	15.5 (10.8)	23.3 (16.5)	6	2	2	2	93.5 (20.6)	−1.13	0.15	0.68	10/12 (83%)
CTRL	37.0 (12.75)	23–64	4/6	n/a	n/a	n/a	n/a	n/a	n/a	114.9 (36.4)	0.68	2.13	1.47	n/a

^a^ According to the ILAE classification 2001.

Abbreviations: CTRL, controls; DNET, Dysembryoplastic Neuroepithelial Tumor; F, females; IQR, interquartile range; LTLE, left temporal lobe epilepsy; M, males; n, number of observations; n/a: not applicableRTLE; RTLE, right temporal epilepsy; SD, standard deviation.

The study was approved by the National Hospital for Neurology and Neurosurgery and the University College London Institute of Neurology Joint Research Ethics Committee and Health Research Authority. Written informed consent according to the Declaration of Helsinki was obtained from all participants.

### 2.2 Neuropsychology

Patients and controls underwent standardized language assessment preoperatively and at the three timepoints postoperatively. Linguistic assessment included:

(1) McKenna Graded Naming Test,^29^ which is a visual confrontation naming assessment (referred to as Picture Naming).
(2) Verbal Fluency assessments using Phonemic Fluency for one letter “S”^30^ (“PF”), and Categorial (Semantic) Fluency using Animal Naming^30^ (“CF”).

### 2.3 Magnetic resonance data acquisition and experimental fMRI task

Functional and structural scans were conducted on a 3 T GE Signa HDx MRI scanner with a 20‐channel head coil. Structural scans utilized T1 contrast, fast‐spoiled gradient echo (FSPGR). Language fMRI used T2×‐weighted gradient echo planar imaging (EPI). Long‐term follow‐up MRI scans were performed on a 3 T GE Discovery MR750 scanner with a 32‐channel head coil, adjusting parameters (Tables S1 and S2). The Verbal fluency (VF) fMRI task assessed linguistic functional activations. Participants covertly generated words starting with a given letter for 20 s (active condition) followed by a 20 s rest block.

### 2.4 Pre‐ and post‐operational data pre‐processing

Pre‐ and postoperative three‐dimensional (3D)‐T1 images were corrected for field bias using advanced normalization tools (ANTs).^31^ Imaging time‐series were realigned to the mean image and time‐corrected with SPM12. Non‐rigid alignment of fMRI scans was performed using EasyReg (FreeSurfer deep‐learning method), aligning first to postoperative T1s for EPI distortion correction, and then to a scanner‐specific Montreal Neurological Institute (MNI) template.^19^ Normalization to MNI space involved non‐rigid transformation of the postoperative T1 to the scanner‐specific template, followed by warping of the time‐series using nearest‐neighbor interpolation. The scanner‐specific template consisted of high‐resolution EPI data of 30 neurotypical CTRL, 15 with left and 15 with right HS. Spatial smoothing was applied in statistical parametric mapping 12 (SPM12) with an 8 mm full width at half maximum (FWHM) Gaussian kernel.

### 2.5 First‐level processing and group contrasts

The generalized linear model (GLM) mapped the hemodynamic response curve onto each experimental condition using boxcar regressors. The regressors were fitted to the time series at each voxel, generating weighted beta images. The resulting model was converted to a *t*‐statistic image, forming the statistical parametric map. Second‐level analysis generated activation maps for each group (LTLE, RTLE, CTRL) separately. This involved computing standard error and average for a contrast estimate, and then assessing statistical significance using separate one‐sample *t* tests for each group. Group contrasts were created for patients at each time point (pre‐operatively, and 4 months‐, 12 months‐, and ~9 years post‐operatively) and at equivalent time points for CTRL.

### 2.6 Statistical analyses of fMRI data

#### 2.6.1 Clinical and neuropsychological data

Raw scores were standardized into z‐scores using aging norms for PF and Picture Naming assessment.^32^, ^33^ Univariate analyses of variance (ANOVAs) were performed at each time point using IBM SPSS Statistics (v29.0) to assess group differences in *z*‐scores, with Bonferroni post hoc tests identifying significant pairwise differences. Mixed‐model ANOVAs tested differences in *z*‐scores across time points, between groups (LTLE, RTLE, CTRL), and group‐timepoint interactions. Age, epilepsy duration (ED), and IQ were correlated (Spearman's *ϱ*) with *z*‐scores at ~9‐years follow‐up time‐point.

Improvement or decline in test scores was calculated and considered clinically significant based on the RCI with a 95% CI^15^, ^34^ The RCI probes meaningful change by adjusting for test reliability and practice effect in a test–retest context.^15^ The practice effects were not corrected for when calculating RCIs between 12 months and ~9 years post‐surgery.

#### 2.6.2 Imaging

##### Language dominance and functional lateralization in different regions of interest (ROIs)

To examine regional plasticity effects, lateralization indices (LIs) quantitatively assessed hemispheric dominance and functional lateralization in various regions of interest (ROIs) using the bootstrap method in the lateralization index toolbox in SPM12 on VF spmT maps. ROIs were defined by anatomic frontal, temporal, and cerebellar masks.^35^ We also report whole‐brain LIs (without cerebellum, which typically shows crossed lateralization). One‐way ANOVAs with repeated measures were conducted to assess whether significant differences existed in the mean LIs of individual participants across the four time points within each group and mask and if the change in LIs across time‐points within each group was significant.

Group activations are shown corrected for multiple comparisons family‐wise error (FWE) at *p* < .05 and visualized in figures at *p* < .001 (uncorrected). Specific flexible factorial (FF) analyses comparing individuals with epilepsy across time‐points against CTRL, and correlations, are reported at a threshold of *p* < .001 (uncorrected), consistent with prior longitudinal, event‐related, and network fMRI studies.^27^, ^36^

##### Relation between voxel‐based change in fMRI activation patterns and the change in language performance in the long‐term post‐surgery

A change (difference) image for each patient was created using ImCalc tool in SPM12. Changes in PF and Picture Naming z‐scores from 12 months to ~9 years post‐surgery were calculated and correlated with changes of fMRI activation between 12 months and ~ 9 years post‐surgery. The change in performance z‐scores were used as continuous regressors in an ANCOVA to investigate brain regions associated with better and worse language performance.

##### Twelve month to 9‐year changes in the VF network

Short‐term plasticity has been explored and reported previously.^11^ We, therefore, concentrated on the longer‐term plasticity changes from 12 months to 9 years postoperatively.

A mixed ANOVA using an FF design in SPM12 was employed to evaluate functional reorganization over time in patient groups, considering changes beyond test–retest observed in CTRL. Different pathologies are related to distinct alterations of the language function in TLE^37^; therefore, we performed a separate sub‐analysis on a homogeneous group of patients with hippocampal sclerosis (HS). A random‐effects analysis was first performed to account for variance between and within subjects. Data from each subject's first‐level analysis was then categorized into three groups: CTRL, LTLE, and RTLE. To evaluate changes in brain activation across sessions, mixed ANOVAs with an FF design were conducted, comparing (1) LTLE vs CTRL and RTLE vs CTRL. Each 2 × 3 ANOVA treated “Group” (LTLE, RTLE, CTRL) as a between‐subject factor and “Condition” (12 months post‐operation, ~9 years post‐operation) as a within‐subject factor.

## 3 RESULTS

### 3.1 Demographic information

There was no significant difference in the proportion of male and female participants in the patient and control groups (two‐sided Fisher exact test). No significant differences were found in age, handedness, or number of TLE with HS in left and right TLE (assessed with Kruskal–Wallis tests). All RTLE patients were right‐handed with left‐dominant language. Two people with LTLE were left‐handed; one had bilateral language representation. In our cohort, 83% (20/24) of patients were seizure‐free ~9 years post‐surgery. Four patients (two LTLE, two RTLE) still experienced daily or weekly seizures; three were in ILAE class 2, and one in ILAE class 3. There was no significant difference in the age at epilepsy onset between non‐HS and HS patients for both LTLE and RTLE (*t* tests), as well as, for separate groups of epilepsy etiologies (ANOVA).

### 3.2 Neuropsychological results

**FIGURE 1:**
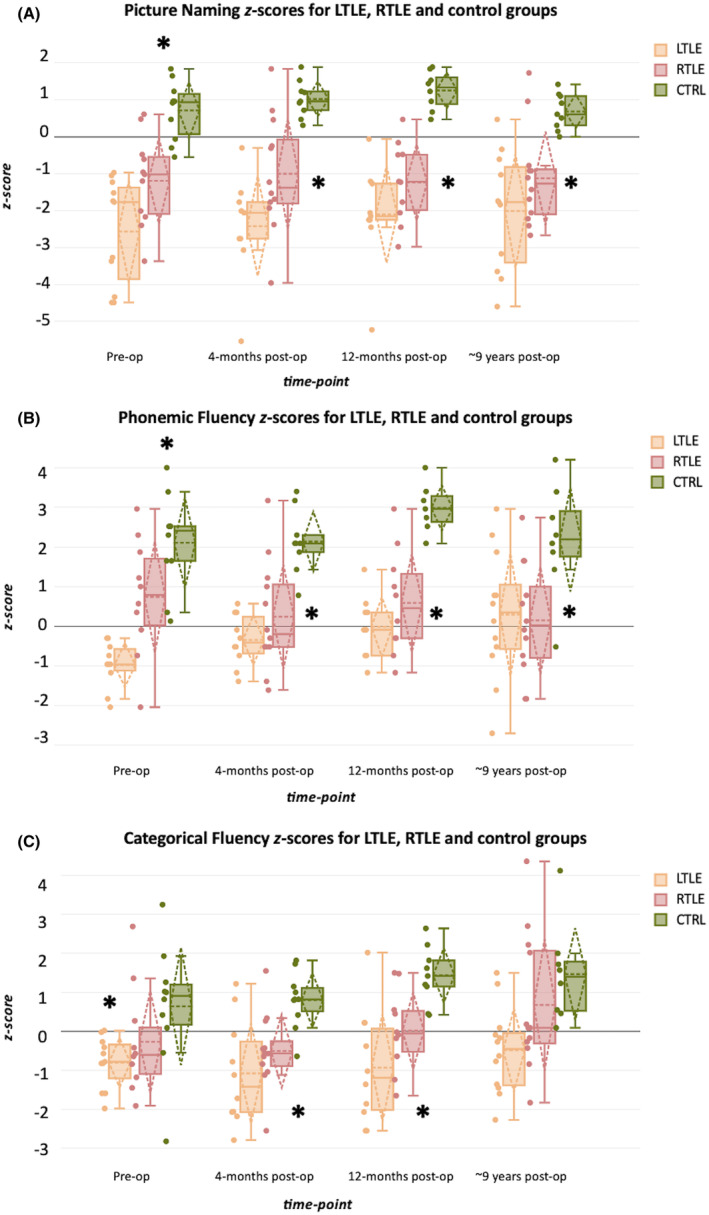
Neuropsychological assessment—*z*‐score distribution for Picture Naming (A), Phonemic Fluency (B), and Categorical (Semantic) Fluency (C) for LTLE, RTLE and CTRL groups across four time‐points. The lower end of the box is the first quartile and the upper end is the third quartile of the data range; the solid line in the box indicates the median, and the dotted line—the mean; the dotted lines represent the SDs; the asterisk “*” above the boxplots indicates significant differences in *z*‐scores across all three groups; an asterisk “*” next to the patient groups’ boxplots (on the right) indicates significant differences between both patient groups’ *z*‐scores and CTRL *z*‐scores; the asterisk “*” on the left of the boxplots indicates significant differences between LTLE and CTRL.

Neuropsychological data for the LTLE, RTLE, and control groups across the four timepoints are shown as boxplots in Figure 1. Mean (SD) values and percentages of patients and controls with clinically significant decline or improvement (based on RCIs) at each timepoint are provided in Table 2. Statistically significant correlations of age, ED, and IQ with *z*‐scores at ~9 years follow‐up are presented in the text after Table 2.

**TABLE 2:** Table of mean *z*‐scores (and SDs) for Picture Naming, Phonemic and Categorical Fluency for LTLE, RTLE, and CTRL, together with the percentages of patients and controls showing clinically significant decline or improvement (based on RCIs) between pre‐surgery and 4 months, 4 and 12 months, and 12 months and ~9 years post‐surgery.

Task	Group	Pre‐surgery	4 Months post‐surgery	12 Months post‐surgery	~9 Years post‐surgery
Picture Naming	*z*‐Scores (Mean ± SD)				
	LTLE (*n* = 12)	−2.57 ± 1.44	−2.42 ± 1.43	−2.1 ± 1.40	−2.01 ± 1.64
	RTLE (*n* = 12)	−1.19 ± 1.22	−1.00 ± 1.57	−1.24 ± 1.09	−1.13 ± 1.32
	CTRL (*n* = 10)	0.71 ± 0.81	1.03 ± 0.51	1.25 ± 0.52	0.68 ± 0.49
	Improved (% participants)				
	LTLE	25%		8%	50%
	RTLE	17%		25%	50%
	CTRL	30%		20%	10%
	Declined (%)				
	LTLE	42%		25%	33%
	RTLE	25%		17%	33%
	CTRL	10%		0%	30%
Phonemic Fluency	*z*‐scores (Mean ± SD)				
	LTLE	−0.98 ± 0.58	−0.35 ± 0.65	0.04 ± 0.77	0.29 ± 1.62
	RTLE	0.74 ± 1.48	0.24 ± 1.39	0.59 ± 1.31	0.20 ± 1.41
	CTRL	2.1 ± 1.21	2.14 ± 0.8	2.71 ± 0.97	2.13 ± 1.35
	Improved (% Participants)				
	LTLE	67%		42%	50%
	RTLE	25%		58%	8%
	CTRL	33%		33%	33%
	Declined (%)				
	LTLE	25%		8%	25%
	RTLE	33%		17%	33%
	CTRL	17%		17%	25%
Categorical Fluency	z‐Scores (Mean ± SD)				
	LTLE	−0.8 ± 0.66	−1.08 ± 1.35	−0.93 ± 1.46	−0.48 ± 1.12
	RTLE	−0.27 ± 1.38	−0.5 ± 0.98	0.02 ± 1.04	0.68 ± 1.76
	CTRL	0.64 ± 1.6	0.8 ± 0.73	1.44 ± 0.74	1.04 ± 1.68
	Improved (% Participants)				
	LTLE	25%		42%	58%
	RTLE	33%		67%	42%
	CTRL	30%		58%	20%
	Declined (%)				
	LTLE	42%		16%	25%
	RTLE	33%		33%	16%
	CTRL	10%		0%	10%

(A) *Picture Naming*:

- Pre‐surgery: Significant group differences in z‐scores were observed among CTRL, LTLE, and RTLE (*F* [2, 29] = 19.82, *p* < .001, *η* ^2^ = 0.58). Post hoc tests showed that all groups differed significantly, with LTLE scoring the lowest.
- Four months post‐surgery: Differences persisted (*F* [2, 30] = 16.06, *p* < .001, *η* ^2^ = 0.54), with both patient groups scoring lower than controls.
- Twelve months post‐surgery: Group differences remained significant (*F* [2, 30] = 21.68, *p* < .001, *η* ^2^ = 0.64), with patients continuing to score lower than controls.
- Approximately nine years post‐surgery: Significant differences continued (*F* [2, 31] = 11.28, *p* < .001, *η* ^2^ = 0.44), with patients maintaining lower scores compared to controls.
- Approximately nine years post‐surgery, correlation with age, ED, and IQ: LTLE showed strong positive correlation between Naming *z*‐scores and IQ (*r* = 0.79, *p* = .004), with RTLE showing a very high correlation (*r* = 0.91, *p* < .001).
- LTLE vs RTLE: There was a significant difference between patient groups in z‐scores across the four time points (*F* [1,3] = 14.13, *p* < .001), with RTLE showing higher scores.

(B) *Phonemic Fluency*:

- Pre‐surgery: A univariate ANOVA revealed significant differences among groups (*F* [2, 29] = 17.88, *p* < .001, *η* ^2^ = 0.57), with LTLE scoring the lowest.
- Four months post‐surgery: Significant differences persisted (*F* [2, 30] = 14.77, *p* < .001, *η* ^2^ = 0.51), with controls outperforming both patient groups.
- Twelve months post‐surgery: Group differences remained significant (*F* [2, 30] = 15.58, *p* < .001, *η* ^2^ = 0.55), with controls scoring higher.
- Approximately nine years post‐surgery: Significant differences were still present (*F* [2, 31] = 5.95, *p* < .001, *η* ^2^ = 0.28), with CTRL scoring higher.
- Longitudinal change: A mixed model ANOVA indicated significant increases in LTLE phonemic fluency *z*‐scores over time (*F* [3, 24] = 4.62, *p* = .011).
- LTLE vs RTLE: There was a significant difference between the patient groups in terms of *z*‐score values across four time‐points, (*F* [1,3] = 5.76, *p* = .02), in which RTLE had higher scores.
- Approximately nine years post‐surgery, correlation with age, ED, and IQ: RTLE age (*r* = −0.66, *p* = .019) and ED (*r* = −0.56, *p* = .05) both showed strong negative correlations with PF *z*‐scores.

(C) *Categorical (Semantic) Fluency*:

- Pre‐surgery: Significant group differences were found (*F* [2, 29] = 3.49, *p* < .001, *η* ^2^ = 0.2), with CTRL differing significantly from LTLE.
- Four months post‐surgery: Differences remained significant (*F* [2, 30] = 8.5, *p* = .001, *η* ^2^ = 0.37), with controls scoring higher than patients.
- Twelve months post‐surgery: ANOVA indicated significant group differences (*F* [2, 30] = 10.47, *p* < .001, *η* ^2^ = 0.45), with controls outperforming patients.
- Approximately nine years post‐surgery: No significant group differences were observed (*F* [2, 31] = 2.87, *p* = .07, *η* ^2^ = 0.17).
- Approximately nine years post‐surgery, correlation with age, ED, and IQ: LTLE showed high, negative correlation between age and CF (*r* = −0.73, *p* = .016).
- LTLE vs RTLE: There was a significant difference between the patient groups in terms of *z*‐score values across four time‐points, (*F* [1,3] = 8.71, *p* = 0.004), in which RTLE had higher scores.

In summary, significant group differences were consistently observed across all cognitive measures, in which LTLE and RTLE patients showed lower scores than CTRL across all time points for Picture Naming and Phonemic Fluency. By 9 years post‐surgery, *z*‐scores for Categorical Fluency among LTLE and RTLE patients were comparable to those of control participants, indicating a convergence in performance in this domain. RTLE had consistently higher scores than LTLE for all three tests. Post‐surgery both groups of patients exhibited recovery (and stability), particularly in the first year, but cognitive performance remained below that of controls. In the long‐term, LTLE patients demonstrated significant improvement in PF, and non‐significant improvements in Naming and CF.

### 3.3 Postoperative language change (pre‐surgery to 4–12 month to ~9 year changes)

(A) *Picture Naming*:

Pre‐operatively to 4 months post‐operatively: 25% of LTLE patients showed improvement, whereas 42% declined; 17% of RTLE patients improved, with 25% declining; in the control group, 30% improved and 10% declined.

Four to twelve months post‐operatively: 8% of LTLE participants improved, with 25% declining; 25% of RTLE participants improved and 17% declined; in the control group, 20% showed improvement, with no declines observed.

Twelve months to ~9 years post‐operatively: 50% of both LTLE and RTLE participants improved, whereas 33% declined; in the control group, 10% improved, and 30% declined.

(B) *Phonemic Fluency*:

Pre‐operatively to 4 months post‐operatively: 67% of LTLE patients improved, whereas 25% declined; 25% of RTLE patients improved, with 33% declining; in the control group, 33% improved and 17% experienced a decline. A two‐tailed *z*‐score test indicated greater a proportion of patients improving in the LTLE group (*z* = 2.05, *p* = .04).

Four to twelve months post‐operatively: 42% of LTLE participants improved, whereas 8% declined; 58% of RTLE participants improved, with 17% declining; in the control group, 33% showed improvement and 17% declined.

Twelve months to ~9 years post‐operatively: 50% of LTLE participants improved, whereas 25% declined; 8% of RTLE participants improved, with 33% declining; in the control group, 33% improved and 25% declined. A two‐tailed *z*‐score test indicated a greater proportion of patients improving in the LTLE group (*z* = 2.25, *p* = .02).

(C) *Categorical Fluency*:

Pre‐operatively to 4 months post‐operatively: 25% of LTLE participants improved, whereas 42% declined; 33% of RTLE participants improved, with 33% declining; in the control group, 30% improved and 10% declined.

Four to twelve months post‐operatively: 42% of LTLE participants improved, whereas 16% declined; 67% of RTLE participants improved, with 33% declining; in the control group, 58% improved, with no declines observed.

Twelve months to ~9 years post‐operatively: 58% of LTLE participants improved, whereas 25% declined; 42% of RTLE participants improved, with 16% declining; in the control group, 20% improved and 10% declined.

In summary, for Picture Naming, both LTLE and RTLE patients initially showed limited improvement. However, significant long‐term gains were observed in both groups, although their performance remained lower compared to controls. In Phonemic Fluency, LTLE patients exhibited a greater proportion of improvement than RTLE patients, particularly in the early post‐operative period, and maintained this advantage over the long term. For Categorical Fluency, both LTLE and RTLE patients made substantial progress, with LTLE patients demonstrating more sustained improvements. The data suggest that although RTLE patients experience greater stability in Picture Naming, LTLE patients show more consistent gains in Phonemic Fluency.

Control subjects demonstrated the highest stability in cognitive performance over time, with only minor cognitive decline observed at 9 years post‐surgery. The improvements noted at 4 and 12 months post‐surgery are likely attributable to practice effects, which were addressed through the use of RCIs. The minor decline observed at ~9 years post‐surgery is presumably a result of test–retest effects from earlier assessments at 4 and 12 months, rather than genuine cognitive deterioration. The mean scores at ~9 years remained comparable to baseline scores recorded pre‐surgery, indicating that the long‐term stability of cognitive function in the control group is maintained.

### 3.4 Imaging results

#### 3.4.1 Group activations

**FIGURE 2:**
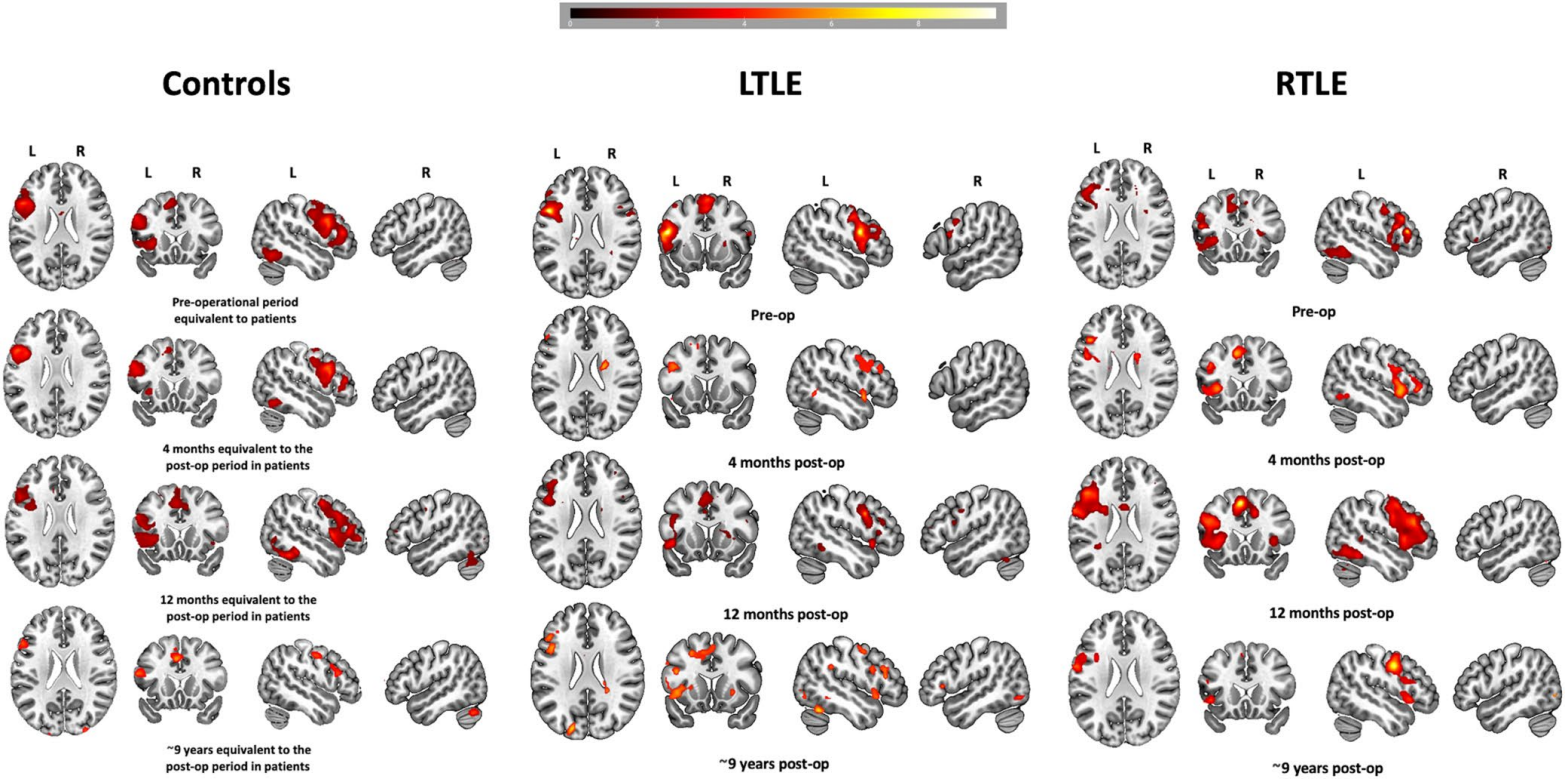
Cross‐sectional, single‐group VF task activation maps for controls and LTLE and RTLE patients at all four time‐points.

Group fMRI activations across four timepoints are shown in Figure 2 and Table S3. In healthy CTRL, cortical activations were consistently left‐lateralized with corresponding right cerebellar activations, with a reduction in blood‐oxygen level–dependent (BOLD) signal at ~9 years follow‐up. In LTLE, dynamic changes in language activation patterns were observed. Preoperative imaging revealed engagement of the right frontal lobe. At 4 months post‐surgery, a reduction in bilateral frontal activation was observed, with a predominance of activation in the left hemisphere (frontal and temporal lobes, including the remnant posterior HC). By 12 months, strong bilateral engagement had re‐emerged, a pattern that persisted in the long‐term follow‐up at 9 years. Language function was supported by bilateral frontal activation (IFG, dorsolateral prefrontal cortex [dlPFC]), bilateral temporal lobes (including the remnant posterior HC, bilateral fusiform), left insula, and bilateral putamen. RTLE patients exhibited a pattern similar to that of the control group, where the activations were left‐dominant across time‐point, with a reduction of BOLD signal at ~9 years follow‐up. These qualitative differences were analyzed quantitatively using an FF analysis, exploring differential lateralization patterns longitudinally in frontal, temporal, and cerebellar regions after left and right ATLR.

#### 3.4.2 Language dominance—Lateralization index

Individual and group mean LIs were calculated for the whole cerebral hemisphere, frontal, temporal, and cerebellar ROIs (inclusive masks used; see Table 3 for group LIs). Left hemisphere dominance (LI > +0.2), bilateral (−0.2 ≤ LI ≤ +0.2), and right hemisphere dominance (LI < −0.2) were defined.^22^ Detailed description of the results can be found in Data S1, Section 2.

**TABLE 3:** Table of lateralization indices calculated in four ROIs for LTLE, RTLE, and CTRL.

Inclusive mask	Time	LTLE	RTLE	CTRL
Whole cerebral hemisphere (mask with no cerebellum)	Pre‐op	0.36	0.55	0.79
	4 months post‐op	0.20	0.45	0.71
	12 months post‐op	0.42	0.57	0.69
	~9 years post‐op	0.18	0.39	0.56
Frontal lobe	Pre‐op	0.60	0.73*	0.75
	4 months post‐op	0.53	0.62*	0.78
	12 months post‐op	0.44	0.65*	0.74
	~9 years post	0.38	0.25*	0.54
Temporal lobe	Pre‐op	0.42*	0.57	0.70
	4 months post	0.45*	0.40	0.68
	12 months post‐op	0.21*	0.49	0.61
	~9 years post‐op	0.18*	0.37	0.56
Cerebellum	Pre‐op	−0.39*	−0.53	−0.59
	4 months post‐op	−0.12*	−0.46	−0.60
	12 months post‐op	−0.63*	−0.54	−0.65
	~9 years post‐op	−0.65*	−0.42	−0.60

^*^ Indicates significant changes over time within a group.

LTLE: At 4 months post‐left ATLR, whole‐brain LIs showed reduced left‐lateralization, with frontal LIs decreasing and temporal indices remaining stable. Bilateral cerebellar activity was observed at this time point. By 12 months, frontal lateralization became less left‐lateralized, with a significant decrease in the temporal region. Whole‐brain lateralization indices non‐significantly increased, and cerebellar activity shifted toward right‐lateralization. Over the long term, frontal and temporal LIs continued to decline, with a significant reduction observed in the temporal region.

RTLE and CTRL: RTLE and CTRL remained stable across three time points (pre‐surgery to 12 months post‐operatively), with non‐significant reductions in LIs across all ROIs over ~9 years, except for a significant decrease in frontal LIs in RTLE.

#### 3.4.3 Long‐term plasticity changes from 12 months to 9 years post‐operatively

A mixed ANOVA using FF design was conducted to study language network plasticity in LTLE and RTLE from 12 months to ~9 years follow‐up, and to compare changes in patients above and beyond those seen in CTRL. Results are reported in Table 4, at *p* < .001 (uncorrected).

**TABLE 4:** Increased and decreased activations in LTLE and RTLE from 12 months to ~9 years post‐surgery (flexible factorial).

Group	Frontal regions	Temporal regions	Extra fronto‐temporal regions
LTLE *n* = 12 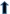	L. IFG, pars opercularis [−58, 8, 6], *p* < .001, [−54, 14, 4], *p* < .001 L. IFG, pars orbitalis [−36, 32, −8], *p* < .001, [−46, 18, −14], *p* < .001 L. IFG, pars triangularis [−39, 33, 39], *p* < .001 L. PreMotor + SuppMotor [−4, 18, 62], *p* < .001 L. AntPFC [−26, 56, 2], *p* < .001	R. Middle Temporal Gyrus [66, −28, −6], *p* < .001, [64, −46, 2], *p* < .001	L. Insula [−40, 12, −4], *p* < .001 L. Putamen [−28, 0, 0], *p* < .001 R. Putamen [24, 9, −12], *p* < .001 R. Cerebellum [34, −74, −26], *p* < .001
LTLE (HS) *n* = 9 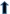	L. IFG, pars opercularis [−58, 6, 6], *p* < .001, [−56, 14, 4], *p* < .001 L. PreMotor + SuppMotor [−48, −4, 50], *p* < .001, [−8, −2, 72], *p* < .001 L. dlPFC [−48, 26, 34], *p* < .001 L. AntPFC [−26, 56, 2], *p* < .001	R. Middle Temporal Gyrus [62, −32, −6], *p* < .001 L. posterior remnant Hippocampus [−22, −16, −14], *p* < .001 R. Superior Temporal Gyrus [50, −24, 4], *p* < .001	R. Putamen [28, 4, −4], *p* < .001 R. Cerebellum [34, −74, −26], *p* < .001 R. Supramarginal Gyrus [62, −32, 46], *p* < .001
LTLE + LTLE (HS) 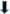	No significant activations at a threshold *p* < .001		
RTLE *n* = 12 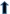	L. IFG, pars opercularis [−56, 12, 8], *p* < .001 L. PreMotor + SuppMotor [−4, 18, 62], *p* < .001 L. AntPFC [−18, 66, −2], *p* < .001	R. Fusiform Gyrus [36, −72, −28], *p* < .001	L. Putamen [−28, −4, 0], *p* < .001
RTLE (HS) *n* = 6 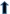	L. IFG, pars opercularis [−58, 6, 8], *p* < .001 L. IFG, pars triangularis [−54, 16, 6], *p* < .001 [−30, 20, 14], *p* < .001 L. PreMotor + SuppMotor [−50, −4, 46], *p* < .001 L. AntPFC [−8, 64, −4], *p* < .001	R. Fusiform Gyrus [40, −70, −16], *p* < .001 L. Caudate [−12, 4, 10], *p* < .001	L. Insula [−40, 12, −4], *p* < .001 L. Putamen [−28, −4, 0], *p* < .001
RTLE 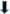		R. Temporal Pole [46, 16, −26], *p* < .001	
RTLE (HS) 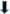		R. Temporal Pole [46, 14, −26], *p* < .001 R. Parahippocampus [38, −23, −20], *p* < .001	

*Note*: Threshold set at voxels attaining *p* < .001, uncorrected. Arrow up indicates the increased activations and arrow down the decreased activations. LTLE and RTLE with (HS) is a subgroup analysis for people with hippocampal sclerosis only.

People with LTLE exhibited increased activations in the left frontal cortex (IFG, pars opercularis, orbitalis, and triangularis) ipsilateral to the side to surgery, left premotor and supplementary motor area (SMA), left anterior prefrontal cortex (AntPFC), right middle temporal gyrus (MTG), left insula, bilateral putamen, and right cerebellum. No significant decreases were found at threshold *p* < .001. For LTLE with HS, the increased activations were similar to the whole group, with additional significant increases in the left dlPFC, left posterior remnant HC, left superior temporal gyrus (STG), and right supramarginal gyrus. Activation increases were also limited to the pars opercularis in the IFG.

In RTLE, increased activations were observed in the left frontal region (IFG, pars opercularis), right fusiform gyrus, left premotor and SMA, left AntPFC, and left putamen, with reductions in the right temporal pole. The RTLE‐HS group showed similar increases to the whole RTLE sample, with additional significant increases in the pars triangularis of IFG, left caudate, and left putamen. The decreased activation was found in right temporal pole and parahippocampus.

#### 3.4.4 Language performance: Long‐term change of VF fMRI activation and change in PF and Picture Naming scores

Change in fMRI activation from 12 months to the long‐term follow‐up (~9 years post‐operatively) was correlated with the change in *z*‐scores between 12 months to ~9 years post‐operatively for PF and Picture Naming. Positive corrections (+) indicated regions that supported better language performance and negative correlations (−) worse language performance (Table 5).

**TABLE 5:** Brain regions that supported better (+) and worse (−) language performance.

Group and test	+	−
LTLE: Verbal Phonemic Fluency	R. Middle Temporal Gyrus [60, −16, −14], *p* < .001 L. Middle Temporal Gyrus [−58, −20, −18], *p* < .001 R. Superior Temporal Gyrus [64, −38, 16], *p* < .001 R. Supramarginal Gyrus [48, −28, 34], *p* < .001 R. Insula [2, 6, 40], *p* < .001 R. Ventral Anterior Cingulum [6, −14, 38], *p* < .001 R. Putamen [20, 18, −4], *p* < .001 L. Angular Gyrus [−38, −76, 32], *p* < .001 R. Fusiform Gyrus [−38, −76, 32], *p* < .001 L. Caudate [−10, 14, −4], *p* < .001	L. Thalamus [−12, −8, 2], *p* < .001
LTLE: Naming	R. IFG (pars triangularis) [42, 24, 0], *p* < .001 R. IFG (pars orbitalis) [32, 36, −16], *p* < .001 R. Parahippocampus [14, −38, −2], *p* < .001 R. Fusiform Gyrus [52, −46, −12], *p* < .001 R. Angular Gyrus [42, −66, 22], *p* < .001 L. remnant hippocampus [−24, −6, −22], *p* < .001 R. Cerebellum [28, −70, −32], *p* < .001	R. AntPFC [10, 64, −6], *p* < .001 L. Dorsal ACC [−10, 34, 18], *p* < .001
RTLE: Verbal Phonemic Fluency	R. Temporal Pole [56, −6, −22], *p* < .001 L. IFG (pars triangularis) [−48, 16, 0], *p* < .001	R. IFG (pars opercularis) [14, 18, 48], *p* < .001 R. Angular Gyrus [44, −68, 46], *p* < .001
RTLE: Naming	No significant activation at *p* < .001	No significant activation at *p* < .001

In LTLE, right temporal activations (MTG, STG, parahippocampus, fusiform), right frontal activations (IFG: pars orbitalis and triangularis), left temporal activation (MTG, remnant HC), and bilateral angular gyrus correlated with improved PF and Naming performance. Improved PF was also associated with activations in the left caudate, right putamen, and right anterior cingulum, whereas improved Naming was linked to the right cerebellum. Conversely, activations in the left thalamus, right AntPFC, and left dorsal anterior cingulate cortex (ACC) correlated negatively with performance changes.

In RTLE, left IFG (pars triangularis) and right temporal pole associated with improvements in PF and Naming function. Right IFG (pars opercularis) and right angular gyrus correlated negatively with performance.

## 4 DISCUSSION

### 4.1 Summary of the main findings

The longitudinal changes in task‐evoked neural activity suggest that People with LTLE increase engagement of the left extra‐temporal and contralateral temporal regions long‐term post‐ATLR, with better language performance being linked to the recruitment of bilateral temporal and extra‐temporal areas. LTLE patients with HS demonstrate more extensive bilateral extra‐temporal and temporal activations (including the remnant posterior HC), indicating more widespread activations compared to those without HS. People with right TLE exhibit plasticity in the left extra‐temporal regions and right temporal regions, with the engagement of these areas correlating with enhanced language performance; whereas the RTLE subgroup with HS showed more left extra‐temporal activations and left temporal region engagement. The findings support the idea that compensatory neuroplasticity underpins sustained improvements in language function long‐term post‐surgery, and highlight the adaptive capacity of the brain's language‐supportive networks in response to temporal lobe resection.^19^ Clinically, this adaptive reorganization can serve as a predictor of better cognitive outcomes, allowing clinicians to tailor rehabilitation strategies to enhance recovery.

LTLE patients showed greater declines in Picture Naming and Categorical Fluency early post‐surgery, whereas RTLE patients had reductions in Phonemic and Categorical Fluency. At the 9‐year follow‐up, both groups achieved cognitive stability, with over 50% of LTLE patients demonstrating language improvement, indicative of sustained neuroplasticity. Factors such as older age, longer epilepsy duration, and limited pre‐operative cognitive reserve were negatively correlated with language performance at ~9 years post‐surgery. These results highlight the distinct and asymmetrical impacts of temporal lobe resections on language function and affirm the potential for long‐term functional improvement, underscoring the importance of considering these factors in pre‐operative counseling.

### 4.2 Seizure outcome

Eighty‐three percent of our patients were seizure‐free ~9 years post‐surgery, slightly exceeding the 70.8% to 74% reported in decade‐long studies,^38^, ^39^ likely due to the smaller sample size in our study. Among the four patients who continued to have seizures, three experienced only auras/focal awake seizures and showed improved language test performance from pre‐surgery to 12 months post‐surgery, maintaining this improvement for up to ~9 years. The reduction in seizures likely contributes to long‐term cognitive stability and improvements observed in these patients, possibly enhancing attention, memory, and overall cognitive function.^40^, ^41^ In contrast, one RTLE patient, who had fewer than 4 days of focal impaired awareness seizures per year, initially improved on neuropsychological tests up to 12 months post‐surgery but subsequently exhibited significant long‐term declines on all three tests. This decline is consistent with research linking ongoing seizures to language deterioration, regardless of seizure side.^37^

### 4.3 Neuropsychology

In our study, language outcomes varied by resection group up to ~9 years post‐surgery, as demonstrated previously in long‐term memory research.^19^ LTLE and RTLE patients had significantly lower Picture Naming and PF scores than CTRL at all time points. For CF, scores were lower in patients up to 12 months post‐surgery but converged with CTRL by ~9 years, indicating recovery. LTLE patients had significantly lower scores than RTLE patients, indicating more severe impairment.

In healthy CTRL, potential practice effects were observed, with notable improvements in language scores between the pre‐operative and the 4‐ to 12‐month follow‐up period. However, this improvement diminished over the long term, with scores showing a decline from the 4‐ to 12‐month period. It is important to note that when comparing long‐term scores to pre‐surgery (baseline) assessments, no significant decline was observed, indicating generally stable cognitive function over time. Similar findings regarding the control group were found in long‐term memory in TLE research.^19^ The findings indicate that the observed changes in the epilepsy groups are likely attributable to the surgical intervention.

#### 4.3.1 Short‐term effect of unilateral resection

Our findings on short‐term postoperative language changes align with prior research.^42^, ^43^, ^44^, ^45^ Notably, although many LTLE patients improved in PF, Picture Naming and CF showed a greater risk of decline, particularly early post‐surgery, with more pronounced decline compared to RTLE. Similar results were reported by Bonelli et al.,^11^ who found that 50% of LTLE patients experienced significant declines in Naming at 4 months post‐surgery. Giovagnoli et al.^46^ also observed initial Naming declines after left ATL, followed by later improvement, as seen in our study at 12 months post‐surgery.

Thirty‐three percent of RTLE patients showed significant declines in Phonemic and Categorical Fluency at 4 months post‐surgery, a higher rate than reported previously.^45^ RTLE patients also exhibited greater RCI‐based declines in PF than in Naming, consistent with prior studies,^11^, ^14^ and higher PF decline compared to LTLE, as also noted by Sherman et al.^45^ Language architecture in the right TLE has been less thoroughly studied overall, despite the frequent occurrence of deficits.^47^ The right hemisphere's supportive role in language processing has been demonstrated in healthy individuals, where greater right‐sided involvement was associated with stronger language abilities.^48^ Foesleitner et al.^49^ found widespread network remodeling in RTLE pre‐ and post‐surgery, implying that a lesion in the right hemisphere can measurably impact language ability. In addition, decreased language function was seen in patients following right‐sided glioma surgery.^50^, ^51^

The lower pre‐operative scores in our RTLE cohort may point to limited cognitive reserve, which might explain their increased susceptibility to post‐surgical decline.^14^, ^19^ Typically, better pre‐operative performance is associated with better post‐operative outcomes.^46^ This is in line with the idea that mental reserve, which depends on the integrity of the epileptogenic zone and unaffected brain regions, plays a crucial role in cognitive outcomes.^14^ All patients who experienced declines also remained on anti‐seizure medication, a factor previously associated with poorer cognitive outcome, especially decreased processing speed.^52^ It is reassuring that PF scores improved at 12 months post‐surgery for RTLE based on RCIs. Given that PF involves time‐limited responses rather than item learning, this improvement is unlikely due to a test–retest effect.^46^

#### 4.3.2 Long‐term effect of unilateral resection

The major focus of this paper was to examine the long‐term effects of unilateral ATLR, also in right ATLR on language function, which have not been thoroughly examined before.^24^ In the current study, both patient groups showed cognitive stability over 9 years postoperatively, although their scores were consistently significantly lower than the scores of the control group. Our longitudinal findings align with prior literature, indicating seizure remission and general cognitive stability <5 years postoperatively in LTLE.^25^, ^26^, ^39^, ^53^

Over 50% of LTLE patients improved on Picture Naming, PF, and CF tests between 12 months and ~9 years post‐surgery, likely due to the gradual release of frontal, posterior temporal, and parietal regions from interference caused by epileptic discharges.^46^ The sustained large improvement in Naming and Fluency tests suggests ongoing brain plasticity long after surgery, as noted previously.^46^

In the RTLE patients, scores for CF and Picture Naming also improved over time (50% and 42% improvement, respectively), whereas PF scores improved at 12 months but then showed a long‐term decline on a group level. This decline was particularly pronounced in patients over the age of 60, aligning with prior research that highlights more significant post‐surgical cognitive decline in older adults.^26^, ^41^, ^43^ Verbal fluency and naming are particularly susceptible to age‐related decline,^43^, ^54^ with these cognitive measures declining by about 1 SD over 30 years.^14^ For example, Galovic et al.^55^ reported that patients with focal epilepsy, predominantly those with a temporal lobe seizure focus, exhibited widespread progressive cortical thinning compared to healthy aging individuals, with peak volume decrease at 5 years after seizure onset. In our study, older age and a longer duration of epilepsy showed a strong negative correlations with long‐term PF *z*‐score. Fleury et al.^19^ also found worse long‐term verbal memory in RTLE patients compared to CTRL after 10 years. Longer epilepsy duration was associated with impaired memory performance, suggesting that extended disease burden disrupts cognitive recovery, even a decade after surgical intervention. From our analysis, one of four CTRL over 60 years of age showed significant decline, whereas the others remained stable, highlighting older age at surgery as a negative prognostic factor for cognitive outcomes, even with seizure freedom. Given the link between longer epilepsy duration and lower PF *z*‐scores,^56^ early surgical intervention may enhance language recovery in patients with drug‐resistant TLE. Cognitive reserve also impacts long‐term outcomes, with better baseline performance predicting superior cognitive function, including in older adults.^14^, ^46^, ^57^ In our study, RTLE patients had (although not statistically significant) lower IQs than both CTRL and LTLE patients, and lower baseline scores than CTRL, which also may explain the reduction seen at ~9 years post‐surgery.

### 4.4 Imaging

#### 4.4.1 Language fMRI in TLE—Postoperative plasticity

##### Short‐term reorganization findings

Short‐term postoperative language network plasticity has been described after both left and right ATLR.^11^, ^17^, ^23^, ^49^, ^58^, ^59^ Following surgery, LTLE patients may exhibit a shift in language activation toward the contralateral hemisphere.^58^ Post‐surgical studies have documented increased bifrontal activation^11^, ^58^ alongside reduced left frontal and left posterior HC activation compared to preoperative levels,^11^, ^49^ and also greater functional connectivity to the homotopic contralateral regions in the right IFG and MFG, with some reports indicating decreased activation in both left and right frontal regions.^17^ In addition, an increase in right anterior temporal lobe activation has been noted.^49^ Left frontal activation was related to better naming scores 4 months after surgery.^11^ Our findings suggest that in LTLE, both pre‐ and 4 months post‐surgery, language networks involved the right frontal hemisphere, with generally reduced bilateral BOLD activation post‐surgery, as shown by second‐level group activations. This is consistent with Bonelli et al.^11^ and Wong et al.^17^ Whole‐brain and cerebellar LIs in our cohort further indicated a more bilateral representation. The reactivation of left temporal regions, including the STG, left posterior remnant HC, and left fusiform at 4 months post‐surgery, accounts for the absence of a significant reduction in temporal LIs. By 12 months post‐surgery, bilateral engagement was more pronounced, correlating with a significant decline in both left frontal and temporal LIs, statistically significant in the temporal ROI. Our short‐term findings for people with RTLE are consistent with those of Wong et al.,^17^ who reported that language activation patterns remained unchanged postoperatively. CTRL showed no change in the short‐term follow‐up and had more left‐lateralized LIs compared to patients, especially in LTLE, consistent with previous findings.^60^

The early post‐operative recruitment of the right hemisphere may be attributed to left‐hemispheric damage and the resultant release of right‐hemisphere function, as suggested previously by Landis et al.^61^ The disinhibited right hemisphere in a result of temporary or permanent damage, may extend its dominance to encompass language functions traditionally considered to be the domain of the left hemisphere.^62^ The disinhibition and compensatory role of the right hemisphere in language recovery following left‐hemispheric injury has been well‐documented in post‐stroke literature. For example, Saur et al.^63^ identified three phases of language recovery, where reduced left‐hemisphere activation is followed by the recruitment of homologous right‐hemisphere regions, facilitating language improvement. A recent meta‐analysis by Wilson and Schenck^20^ reinforced this, concluding that although the right hemisphere is important in supporting recovery when the left hemisphere is compromised, optimal language outcomes also depend on reintegration of left temporal regions into the functional network. Our data support the notion of early right‐hemisphere engagement post LTLE surgery, emphasizing the importance of bilateral, extra‐temporal involvement in post‐surgical language reorganization.

##### Long‐term reorganization findings

Long‐term language reorganization in left and right TLE, assessed via task‐based fMRI, has not been thoroughly studied, with the longest median follow‐up being 6 months.^23^, ^49^ Our FF analysis revealed that, over the long term, LTLE patients exhibited increased left extra‐temporal and right temporal activations. These changes account for the observed reduction in left‐lateralized temporal LIs. Notably, the LTLE subgroup with HS demonstrated more pronounced increases in both left and right extra‐temporal activations and bilateral temporal activations (including the remnant posterior HC), indicating more widespread activations compared to those without HS. Extra‐temporal engagement suggests compensatory plasticity, wherein highly specialized cortical regions are “recruited” in an effort to restore functions disrupted by epilepsy and surgical intervention.^19^ These findings support previous research indicating ipsilateral recruitment involving the left posterior hippocampal remnant in memory tasks in the short‐term,^27^, ^64^ and the engagement of the contralateral neocortical regions involved in the long term.^27^ The results also suggest that neuroplasticity persists long‐term following ATLR, as indicated by increased engagement of the left extratemporal network, a pattern that parallels the increased activation of the left frontal lobe observed in long‐term post‐stroke studies.^21^

RTLE showed less extra‐temporal plasticity change than LTLE, like previously shown in memory research.^27^ There was an increase in left extra‐temporal regions and right fusiform gyrus and decrease in the right temporal regions. The sub‐group of people with HS showed more left extra‐temporal activations and left temporal region engagement and decrease in right temporal regions (including right parahippocampus). Thus, similarly to the LTLE, the findings indicate ongoing neuroplasticity within the RTLE cohort. The findings illustrate that language function is not confined to the dominant hemisphere but comprises an extended network that includes contralateral structures, and damage and/or surgery in the non‐dominant hemisphere may still result in reorganization.^11^

#### 4.4.2 Brain regions supporting better cognitive outcome ~9 years after surgery

Our findings suggest that long‐term plasticity following ATLR may support improvements in language function, with distinct patterns of engagement between left and right TLE patients. In TLE research, the contralateral contribution of language areas was a good prognostic factor for postoperative language ability.^65^ We showed that in LTLE, engagement of both left and right extra‐temporal and bilateral temporal regions associated with improvements in language function, whereas in right TLE, primary left extra‐temporal regions associated with better function. This aligns with the work of Fleury et al.,^19^ who showed that 10 years after surgery, the successful verbal and visual memory‐encoding networks in ATLR engaged bilateral neocortical engagement and bilateral temporal regions. Notably, increased contralesional extra‐temporal engagement was found to be adaptive, with greater activation of the left inferior frontal cortex correlating with better verbal memory outcomes in people with right ATLR. Our results similarly suggest that in language, engagement of the bilateral extra‐temporal and temporal regions remain important in sustaining an efficient, long‐term postoperative neural network. Consequently, both groups of patients in our cohort tend to show progressive improvements in language function over time.

#### 4.4.3 TLE and cerebellum

The cerebellum has a role in cognitive processing.^8^ Focal epileptogenic lesions like HS can cause widespread cerebellar degeneration, especially in drug‐resistant TLE,^66^ altering cerebellar activity observed on MRI. A recent study using functional connectivity identified cerebellar language network dysfunction in epilepsy patients.^12^ The authors found significant reorganization in the right cerebellum, suggesting that abnormal coordination with the anterior cingulate may contribute to naming dysfunction, whereas disconnection with the left IFG may underlie language fluency deficits. Gelinas et al.^9^ highlighted the importance of considering ipsilateral cerebellar activation concerning cerebral language activation during language function assessments. We showed that in people with LTLE, cerebellar language lateralization varied from pre‐surgery to ~9 years post‐surgery, unlike in RTLE or CTRL groups where LIs remained stable. Following left ATLR, initially right‐dominant activity shifted to bilateral and eventually a strong right‐lateralized pattern in the long‐term. These results align with prior research indicating cerebellar activation changes within language networks in TLE.

## 5 STRENGTHS AND LIMITATIONS

We present a unique longitudinal design spanning many years, in which the same participants have data acquired at four time‐points compared to the same control population. Despite a limited sample size, we showed statistically significant network changes and correlations. Scanner types differed across follow‐ups; however, all analyses evaluated differences in patients relative to controls, mitigating scanner‐related effects.

Due to the large variety of medications prescribed, individual medication effects could not be assessed comprehensively. Five patients (three LTLE, two RTLE) discontinued antiseizure medications 12 months after surgery, whereas LTLE and RTLE groups were matched in medication usage. Although most patients were seizure‐free, the impact of ongoing seizures on neuronal plasticity could not be specifically evaluated in this study.

## 6 CONCLUSION

Our study represents the first investigation into the long‐term (>6 month) task‐based functional reorganization of language network plasticity following epilepsy surgery. We demonstrate that the brain remains capable of neuroplastic adaptations for up to ~9 years after left and right ATLR. Effective language recovery was associated with sustained engagement of bilateral temporal and extra‐temporal regions, highlighting the importance of extensive neural networks in postoperative functionality. The results challenge the traditional view of the localized impact of unilateral resection, advocating for a broader perspective that recognizes the adaptive reorganization of neural networks beyond the dominant hemisphere. This adaptive reorganization can serve as a predictor of better cognitive outcomes, enabling clinicians to develop targeted rehabilitation strategies to optimize recovery.

Both LTLE and RTLE patients demonstrated stability and/or general improvements in language functions up to 9 years post‐surgery, although prolonged epilepsy duration, older age, and lower pre‐operative cognitive reserve adversely affected recovery. These insights are crucial for refining post‐surgical rehabilitation strategies and adjusting long‐term recovery expectations. They highlight the significance of early surgical intervention and pre‐operative cognitive reserve in optimizing outcomes and underscore the necessity for individualized pre‐operative counseling and rehabilitation planning. Understanding the asymmetric effects of temporal lobe resections and the potential for sustained improvement emphasizes the importance of considering patient‐specific factors in therapeutic approaches.

## AUTHOR CONTRIBUTIONS

M.K.S. formulated the study design. Data were acquired by M.N.F., M.K.S., G.P.W., and Andrea Hill (radiographer). M.S. carried out the image processing and statistical analyses of neuropsychological and imaging data, performed additional explorative analyses, interpreted the data, wrote the manuscript, and prepared all the supporting material. M.S., L.P.B., and M.N.F. contributed to imaging data processing. M.S., M.K.S., G.d'A., and J.S.D. contributed to data interpretation and manuscript preparation. S.B. and M.N.F. acquired neuropsychological data. M.K.S., D.P.C., G.d'A., and J.S.D. supervised data analysis, interpretation, and manuscript preparation.

## FUNDING INFORMATION

This project was supported by National Institute for Health Research University College London Hospitals Biomedical Research Centre (UCLH/UCL BRC) and Medical Research Council (ID MR/X031039/1) who support M.K.S. This project is also supported by the Wellcome Trust Innovation Program (218380). M.K.S. and M.F. are also supported by the Epilepsy Society. G.P.W. was supported by the Medical Research Council (G0802012, MR/M00841X/1).

## CONFLICT OF INTEREST STATEMENT

The authors declare no conflicts of interest.

## ETHICS STATEMENT

The study was approved by the National Hospital for Neurology and Neurosurgery and the University College London Institute of Neurology Joint Research Ethics Committee and Health Research Authority. Written informed consent according to the Declaration of Helsinki was obtained from all participants. We confirm that we have read the Journal's position on issues involved in ethical publication and affirm that this report is consistent with those guidelines.

## Supporting information


Data S1.

## Data Availability

Anonymized data for replication of this study are available from the corresponding author upon reasonable request.

## References

[1] Engel J Jr . A proposed diagnostic scheme for people with epileptic seizures and with epilepsy: report of the ILAE Task Force on Classification and Terminology. Epilepsia. 2001;42(6):796–803. 11422340 10.1046/j.1528-1157.2001.10401.x

[2] Trimmel K , Caciagli L , Xiao F , van Graan LA , Koepp MJ , Thompson PJ , et al. Impaired naming performance in temporal lobe epilepsy: language fMRI responses are modulated by disease characteristics. J Neurol. 2021;268:147–160. 32747979 10.1007/s00415-020-10116-x PMC7815622

[3] Balter S , Lin G , Leyden K , Paul B , McDonald C . Neuroimaging correlates of language network impairment and reorganization in temporal lobe epilepsy. Brain Lang. 2019;193:31–44. 27393391 10.1016/j.bandl.2016.06.002 PMC5215985

[4] Allone C , Buono VL , Corallo F , Pisani LR , Pollicino P , Bramanti P , et al. Neuroimaging and cognitive functions in temporal lobe epilepsy: a review of the literature. J Neurol Sci. 2017;381:7–15. 28991719 10.1016/j.jns.2017.08.007

[5] Dym RJ , Burns J , Freeman K , Lipton ML . Is functional MR imaging assessment of hemispheric language dominance as good as the Wada test?: a meta‐analysis. Radiology. 2011;261(2):446–455. 21803921 10.1148/radiol.11101344

[6] Gaillard WD , Balsamo L , Xu B , McKinney C , Papero PH , Weinstein S , et al. fMRI language task panel improves determination of language dominance. Neurology. 2004;63(8):1403–1408. 15505156 10.1212/01.wnl.0000141852.65175.a7

[7] Stoodley CJ . The cerebellum and cognition: evidence from functional imaging studies. Cerebellum. 2012;11:352–365. 21373864 10.1007/s12311-011-0260-7

[8] Mariën P , Ackermann H , Adamaszek M , Barwood CH , Beaton A , Desmond J , et al. Consensus paper: language and the cerebellum: an ongoing enigma. Cerebellum. 2014;13:386–410. 24318484 10.1007/s12311-013-0540-5 PMC4090012

[9] Gelinas JN , Fitzpatrick KP , Kim HC , Bjornson BH . Cerebellar language mapping and cerebral language dominance in pediatric epilepsy surgery patients. NeuroImage Clin. 2014;6:296–306. 25379442 10.1016/j.nicl.2014.06.016 PMC4215475

[10] Janszky J , Mertens M , Janszky I , Ebner A , Woermann FG . Left‐sided interictal epileptic activity induces shift of language lateralization in temporal lobe epilepsy: an fMRI study. Epilepsia. 2006;47(5):921–927. 16686658 10.1111/j.1528-1167.2006.00514.x

[11] Bonelli SB , Thompson PJ , Yogarajah M , Vollmar C , Powell RHW , Symms MR , et al. Imaging language networks before and after anterior temporal lobe resection: results of a longitudinal fMRI study. Epilepsia. 2012;53(4):639–650. 22429073 10.1111/j.1528-1167.2012.03433.x PMC4471632

[12] Pang L , Fan B , Chen Z , Chen Z , Lv C , Zheng J . Disruption of cerebellar–cerebral functional connectivity in temporal lobe epilepsy and the connection to language and cognitive functions. Front Neurosci. 2022;16:871128. 35837122 10.3389/fnins.2022.871128 PMC9273908

[13] De Tisi J , Bell GS , Peacock JL , McEvoy AW , Harkness WF , Sander JW , et al. The long‐term outcome of adult epilepsy surgery, patterns of seizure remission, and relapse: a cohort study. Lancet. 2011;378(9800):1388–1395. 22000136 10.1016/S0140-6736(11)60890-8

[14] Helmstaedter C , Kurthen M , Lux S , Reuber M , Elger CE . Chronic epilepsy and cognition: a longitudinal study in temporal lobe epilepsy. Ann Neurol. 2003;54(4):425–432. 14520652 10.1002/ana.10692

[15] Baxendale S , Thompson P . The association of cognitive phenotypes with postoperative outcomes after epilepsy surgery in patients with temporal lobe epilepsy. Epilepsy Behav. 2020;112:107386. 32911298 10.1016/j.yebeh.2020.107386

[16] Rosenberger L , Zeck J , Berl M , Moore EN , Ritzl EK , Shamim S , et al. Interhemispheric and intrahemispheric language reorganization in complex partial epilepsy. Neurology. 2009;72(21):1830–1836. 19470965 10.1212/WNL.0b013e3181a7114b PMC2690987

[17] Wong S , Jong L , Bandur D , Bihari F , Yen YF , Takahashi AM , et al. Cortical reorganization following anterior temporal lobectomy in patients with temporal lobe epilepsy. Neurology. 2009;73(7):518–525. 19687453 10.1212/WNL.0b013e3181b2a48e PMC2730795

[18] da Silva NM , Forsyth R , McEvoy A , Miserocchi A , de Tisi J , Vos SB , et al. Network reorganization following anterior temporal lobe resection and relation with post‐surgery seizure relapse: a longitudinal study. NeuroImage Clin. 2020;27:102320. 32623138 10.1016/j.nicl.2020.102320 PMC7334605

[19] Fleury MN , Binding LP , Taylor P , Xiao F , Giampiccolo D , Caciagli L , et al. Predictors of long‐term memory and network connectivity 10 years after anterior temporal lobe resection. Epilepsia. 2024. 10.1111/epi.18058 PMC7619481 38990127

[20] Wilson SM , Schneck SM . Neuroplasticity in post‐stroke aphasia: a systematic review and meta‐analysis of functional imaging studies of reorganization of language processing. Neurobiol Lang. 2020;2(1):22–82. 10.1162/nol_a_00025 PMC8057712 33884373

[21] Nenert R , Allendorfer JB , Martin AM , Banks C , Vannest J , Holland SK , et al. Longitudinal fMRI study of language recovery after a left hemispheric ischemic stroke. Restor Neurol Neurosci. 2018;36(3):359–385. 29782329 10.3233/RNN-170767

[22] Trimmel K , van Graan LA , Gonzálvez GG , Haag A , Caciagli L , Vos SB , et al. Naming fMRI predicts the effect of temporal lobe resection on language decline. Ann Clin Transl Neurol. 2019;6(11):2186–2196. 31578819 10.1002/acn3.50911 PMC6856622

[23] Crow AJ , Thomas A , Rao Y , Beloor‐Suresh A , Weinstein D , Hinds WA , et al. Task‐based functional magnetic resonance imaging prediction of postsurgical cognitive outcomes in temporal lobe epilepsy: a systematic review, meta‐analysis, and new data. Epilepsia. 2023;64(2):266–283. 36522799 10.1111/epi.17475 PMC9944224

[24] Alexandratou I , Patrikelis P , Messinis L , Alexoudi A , Verentzioti A , Stefanatou M , et al. Long‐term neuropsychological outcomes following temporal lobe epilepsy surgery: an update of the literature. MDPI. 2021;9:1156. 10.3390/healthcare9091156 PMC8466433 34574930

[25] Andersson‐Roswall L , Engman E , Samuelsson H , Malmgren K . Cognitive outcome 10 years after temporal lobe epilepsy surgery: a prospective controlled study. Neurology. 2010;74(24):1977–1985. 20548042 10.1212/WNL.0b013e3181e39684

[26] Baxendale S , Thompson PJ , Duncan JS . Neuropsychological function in patients who have had epilepsy surgery: a long‐term follow‐up. Epilepsy Behav. 2012;23(1):24–29. 22100066 10.1016/j.yebeh.2011.10.021

[27] Sidhu MK , Stretton J , Winston GP , McEvoy AW , Symms M , Thompson PJ , et al. Memory network plasticity after temporal lobe resection: a longitudinal functional imaging study. Brain. 2016;139(2):415–430. 26754787 10.1093/brain/awv365 PMC4805088

[28] Wieser HG , Blume WT , Fish D , Goldensohn E , Hufnagel A , King D , et al. ILAE Commission Report. Proposal for a new classification of outcome with respect to epileptic seizures following epilepsy surgery. Epilepsia. 2001;42(2):282–286. 11240604

[29] Mckenna P , Warrington EK . Graded Naming Test: Manual. England: NFER‐Nelson; 1983.

[30] Newcombe F . Missile wounds of the brain: a study of psychological deficits. Oxford: Oxford University Press; 1969.

[31] Binding LP , Dasgupta D , Taylor PN , Thompson PJ , O'Keeffe AG , de Tisi J , et al. Contribution of white matter fiber bundle damage to language change after surgery for temporal lobe epilepsy. Neurology. 2023;100(15):e1621–e1633. 10.1212/wnl.0000000000206862 36750386 PMC10103113

[32] Avants BB , Tustison N , Song G . Advanced normalization tools (ANTS). Insight J. 2009;2(365):1–35.

[33] Murphy P , Chan E , Mo S , Cipolotti L . A new revised Graded Naming Test and new normative data including older adults (80–97 years). J Neuropsychol. 2020;14(3):449–466. 31599124 10.1111/jnp.12194

[34] Tombaugh TN , Kozak J , Rees L . Normative data stratified by age and education for two measures of verbal fluency: FAS and animal naming. Arch Clin Neuropsychol. 1999;14(2):167–177. 14590600

[35] Tzourio‐Mazoyer N , Landeau B , Papathanassiou D , Crivello F , Etard O , Delcroix N , et al. Automated anatomical labeling of activations in SPM using a macroscopic anatomical parcellation of the MNI MRI single‐subject brain. NeuroImage. 2002;15(1):273–289. 11771995 10.1006/nimg.2001.0978

[36] Fleury M , Buck S , Binding LP , Caciagli L , Vos SB , Winston GP , et al. Episodic memory network connectivity in temporal lobe epilepsy. Epilepsia. 2022;63(10):2597–2622. 35848050 10.1111/epi.17370 PMC9804196

[37] Foesleitner O , Nenning K‐H , Bartha‐Doering L , Baumgartner C , Pataraia E , Moser D , et al. Lesion‐specific language network alterations in temporal lobe epilepsy. Am J Neuroradiol. 2020;41(1):147–154. 31896570 10.3174/ajnr.A6350 PMC6975318

[38] Elsharkawy AE , Alabbasi AH , Pannek H , Oppel F , Schulz R , Hoppe M , et al. Long‐term outcome after temporal lobe epilepsy surgery in 434 consecutive adult patients. J Neurosurg. 2009;110(6):1135–1146. 19025359 10.3171/2008.6.JNS17613

[39] Mohan M , Keller S , Nicolson A , Biswas S , Smith D , Osman Farah J , et al. The long‐term outcomes of epilepsy surgery. PLoS One. 2018;13(5):e0196274. 29768433 10.1371/journal.pone.0196274 PMC5955551

[40] Baxendale S . The impact of epilepsy surgery on cognition and behavior. Epilepsy Behav. 2008;12(4):592–599. 18299253 10.1016/j.yebeh.2007.12.015

[41] Helmstaedter C , Elger C , Vogt V . Cognitive outcomes more than 5 years after temporal lobe epilepsy surgery: remarkable functional recovery when seizures are controlled. Seizure. 2018;62:116–123. 30359865 10.1016/j.seizure.2018.09.023

[42] Trimmel K , Vos SB , Caciagli L , Xiao F , van Graan LA , Winston GP , et al. Decoupling of functional and structural language networks in temporal lobe epilepsy. Epilepsia. 2021;62(12):2941–2954. 34642939 10.1111/epi.17098 PMC8776336

[43] Thompson P , Baxendale S , McEvoy A , Duncan J . Cognitive outcomes of temporal lobe epilepsy surgery in older patients. Seizure. 2015;29:41–45. 26076843 10.1016/j.seizure.2015.03.017

[44] Ives‐Deliperi VL , Butler JT . Naming outcomes of anterior temporal lobectomy in epilepsy patients: a systematic review of the literature. Epilepsy Behav. 2012;24(2):194–198. 22569529 10.1016/j.yebeh.2012.04.115

[45] Sherman EM , Wiebe S , Fay‐McClymont TB , et al. Neuropsychological outcomes after epilepsy surgery: systematic review and pooled estimates. Epilepsia. 2011;52(5):857–869. 21426331 10.1111/j.1528-1167.2011.03022.x

[46] Giovagnoli A , Parente A , Didato G , Tellez‐Zenteno J , Metcalfe A , Hernandez‐Ronquillo L , et al. The course of language functions after temporal lobe epilepsy surgery: a prospective study. Eur J Neurol. 2016;23(12):1713–1721. 27529582 10.1111/ene.13113

[47] Bartha‐Doering L , Trinka E . The interictal language profile in adult epilepsy. Epilepsia. 2014;55(10):1512–1525. 25110150 10.1111/epi.12743

[48] van Ettinger‐Veenstra HM , Ragnehed M , Hällgren M , Karlsson T , Landtblom AM , Lundberg P , et al. Right‐hemispheric brain activation correlates to language performance. NeuroImage. 2010;49(4):3481–3488. 19853040 10.1016/j.neuroimage.2009.10.041

[49] Foesleitner O , Sigl B , Schmidbauer V , Nenning KH , Pataraia E , Bartha‐Doering L , et al. Language network reorganization before and after temporal lobe epilepsy surgery. J Neurosurg. 2020;134(6):1694–1702. 32619977 10.3171/2020.4.JNS193401

[50] Vilasboas T , Herbet G , Duffau H . Challenging the myth of right nondominant hemisphere: lessons from corticosubcortical stimulation mapping in awake surgery and surgical implications. World Neurosurg. 2017;103:449–456. 28419879 10.1016/j.wneu.2017.04.021

[51] Klein M , Duffau H , De Witt Hamer PC . Cognition and resective surgery for diffuse infiltrative glioma: an overview. J Neuro‐Oncol. 2012;108:309–318. 10.1007/s11060-012-0811-x PMC3351615 22362370

[52] Ortinski P , Meador KJ . Cognitive side effects of antiepileptic drugs. Epilepsy Behav. 2004;5:60–65. 10.1016/j.yebeh.2003.11.008 14725848

[53] Forthoffer N , Brissart H , Tyvaert L , Maillard L . Long‐term cognitive outcomes in patient with epilepsy. Rev Neurol. 2020;176(6):448–455. 32414533 10.1016/j.neurol.2020.04.012

[54] Machado A , Barroso J , Molina Y , Nieto A , Díaz‐Flores L , Westman E , et al. Proposal for a hierarchical, multidimensional, and multivariate approach to investigate cognitive aging. Neurobiol Aging. 2018;71:179–188. 30149289 10.1016/j.neurobiolaging.2018.07.017

[55] Galovic M , van Dooren VQ , Postma TS , Vos SB , Caciagli L , Borzì G , et al. Progressive cortical thinning in patients with focal epilepsy. JAMA Neurol. 2019;76(10):1230–1239. 31260004 10.1001/jamaneurol.2019.1708 PMC6604082

[56] Giovagnoli AR . Characteristics of verbal semantic impairment in left hemisphere epilepsy. Neuropsychology. 2005;19(4):501–508. 16060825 10.1037/0894-4105.19.4.501

[57] Chapin JS , Busch RM , Silveira DC , Wehner T , Naugle RI , Ferguson L , et al. Memory performance in older adults before and after temporal lobectomy for pharmacoresistant epilepsy. Clin Neuropsychol. 2013;27(8):1316–1327. 24159928 10.1080/13854046.2013.850535

[58] Backes W , Deblaere K , Vonck K , Kessels AG , Boon P , Hofman P , et al. Language activation distributions revealed by fMRI in post‐operative epilepsy patients: differences between left‐and right‐sided resections. Epilepsy Res. 2005;66(1–3):1–12. 16118045 10.1016/j.eplepsyres.2005.06.007

[59] Helmstaedter C , Fritz N , Pérez PG , Elger C , Weber B . Shift‐back of right into left hemisphere language dominance after control of epileptic seizures: evidence for epilepsy driven functional cerebral organization. Epilepsy Res. 2006;70(2–3):257–262. 16624525 10.1016/j.eplepsyres.2006.03.005

[60] Rosazza C , Ghielmetti F , Minati L , Vitali P , Giovagnoli AR , Deleo F , et al. Preoperative language lateralization in temporal lobe epilepsy (TLE) predicts peri‐ictal, pre‐and post‐operative language performance: an fMRI study. NeuroImage Clin. 2013;3:73–83. 24179851 10.1016/j.nicl.2013.07.001 PMC3807502

[61] Landis T , Regard M , Graves R , Goodglass H . Semantic paralexia: a release of right hemispheric function from left hemispheric control? Neuropsychologia. 1983;21(4):359–364. 6621864 10.1016/0028-3932(83)90022-2

[62] Taylor KI , Regard M . Language in the right cerebral hemisphere: contributions from reading studies. Phys Ther. 2003;18(6):257–261. 10.1152/nips.01454.2003 14614161

[63] Saur D , Lange R , Baumgaertner A , Schraknepper V , Willmes K , Rijntjes M , et al. Dynamics of language reorganization after stroke. Brain. 2006;129(6):1371–1384. 16638796 10.1093/brain/awl090

[64] Bonelli SB , Thompson PJ , Yogarajah M , Powell RHW , Samson RS , McEvoy AW , et al. Memory reorganization following anterior temporal lobe resection: a longitudinal functional MRI study. Brain. 2013;136(6):1889–1900. 23715092 10.1093/brain/awt105 PMC3673465

[65] Chang Y‐HA , Kemmotsu N , Leyden KM , Kucukboyaci NE , Iragui VJ , Tecoma ES , et al. Multimodal imaging of language reorganization in patients with left temporal lobe epilepsy. Brain Lang. 2017;170:82–92. 28432987 10.1016/j.bandl.2017.03.012 PMC5507363

[66] Streng ML , Froula JM , Krook‐Magnuson E . The cerebellum's understated role and influences in the epilepsies. Neurobiol Dis. 2023;183:106160. 37209926 10.1016/j.nbd.2023.106160

