# Supplementary material for: Long‐term neuroplasticity in language networks after anterior temporal lobe resection
Source: Epilepsia. 2024 Nov 6;66(1):207–25. doi: 10.1111/epi.18147 (PMC11742647; doi:10.1111/epi.18147)
Supplement: Supplementary file 1 — Data S1. [file EPI-66-207-s001.docx]

**Supplementary material**

1. **Supplementary Tables**

Supplementary Table 1. **Functional scanning parameters for pre-surgery assessment and shortly after surgery (4 and 12 months).**

|  | **TR**  **(ms)** | **TE** | **Slice thickness (mm)** | **Slice gap (mm)** | **Slices per volume** | **FOV** |
| --- | --- | --- | --- | --- | --- | --- |
| **fMRI (VF)** | 2750 | 25 | 2.5 | 0.3 | 36 | 24x24 |

Supplementary Table 2. **Functional scanning parameters for long-term follow up (9 years).**

|  | **TR**  **(ms)** | **TE** | **Slice thickness (mm)** | **Slice gap (mm)** | **Slices per volume** | **FOV** |
| --- | --- | --- | --- | --- | --- | --- |
| **fMRI (VF)** | 2750 | 22 | 2.4 | 0.1 | 50 | 24x24 |

Supplementary Table 3. **Whole brain activations in control subjects, LTLE patients and RTLE patients during Verbal Fluency fMRI tasks showed corrected for multiple comparisons (FWE) p < 0.05**.

| FWE  *p<0.05* | **Pre-op** | **4 months**  **post-op** | **12 months**  **post-op** | | | **9 years**  **post-op** |
| --- | --- | --- | --- | --- | --- | --- |
| **LTLE** | L. IFG (Pars opercularis)  *[-48 12 24], p<0.000*  R. IFG (Pars opercularis)  *[56 10 20], p<0.000*  *[44 16 24], p<0.000*  *R. IFG (Pars orbitalis)*  *[54 18 -8], p<0.000*  L. PreMot+ SuppMotor Area  *[-45 6 42], p<0.000*  R. PreMot+ SuppMotor Area  *[56 2 36], p<0.000*  L. Cerebellum  *[-4 -60 -28], p<0.000*  R. Insula  *[34 26 -2], p<0.000* | L. IFG (Pars opercularis)  [-33 9 27], p<0.000  L. remnant posterior hippocampus  [-30 -30 -9], p<0.000  L. dlPFC  [-48 33 30], *p<0.000*  L. Fusiform Gyrus  [-39 -42 -15], *p<0.000*  L. Superior Temporal Gyrus  [-51 9 -6], *p<0.000*  L. Putamen  [-27 3 0], p<0.000  R. IFG (Pars opercularis)  [63 15 18], p<0.000 | | L. PreMot+ SuppMotor Area  [-48 6 32], p<0.000  R. IFG (Pars opercularis)  [50 10 18], p<0.000  L. dlPFC  [-36 32 22], p<0.000  R. dlPFC  [38 44 26], p<0.000  L. remnant posterior hippocampus  [-28 -22 -12], p<0.000  L. AntPFC  [-26 58 18], p<0.000  R. PreMot+ SuppMotor Area  [48 0 32], p<0.000  Right Insula  [30 20 10], p<0.000  Left Insula  [30 20 10], p<0.000  L. Fusiform  [-50 -46 -14], p<0.000  R. Fusiform  [40 -44 -26], p<0.000  L. Putamen  [-31 -4 -7], p<0.000 | | L. Insula  [-38 18 2], p<0.000  L. IFG (Pars opercularis)  *[-48 26 24], p<0.000*  R. IFG (Pars triangularis)  *[50 24 4], p<0.000*  L. remnant posterior hippocampus  [-20 -14 -14], p<0.000  L. Fusiform  *[-48 -56 -26], p<0.000*  L. dlPFC  [-46 28 16], p<0.000  L. PreMot+ SuppMotor Area  *[-26 12 46], p<0.000*  L. Putamen  *[-24 10 0], p<0.000*  R. Putamen  *[24 18 0], p<0.000*  R. Fusiform  *[50 24 4], p<0.000* |
| **RTLE** | L. IFG (Pars opercularis)  *[-48 18 20], p<0.000*  L. dlPFC  [-48 28 30], *p<0.000*  L. PreMot+ SuppMotor Area  *[-8 8 64], p<0.000*  *L. AntPFC*  *[-34 42 26], p<0.000*  L. Fusiform  [-20 -32 -18], *p<0.000*  R. Parahippocampus  [18 -26 -18], *p<0.000* | L. IFG (Pars opercularis)  *[-42 24 21], p<0.000*  L. IFG (Pars triangularis)  *[-48 33 0], p<0.000*  L. PreMot+ SuppMotor Area  *[-3 6 66], p<0.000*  Left Insula  *[-33 21 0], p<0.000*  L. Supramarginal Gyrus  *[-30 -39 42], p<0.000* | | L. IFG (Pars opercularis)  *[-48 24 24], p<0.000*  L. PreMot+ SuppMotor Area  *[-6 6 66], p<0.000*  L. Insula  *[-39 3 6], p<0.000*  L. Supramarginal Gyrus  *[-39 -45 42], p<0.000* | | L. IFG (Pars opercularis)  *[-57 9 6], p<0.000*  L. PreMot+ SuppMotor Area  *[-51 -3 39], p<0.000* |
| **CTRL** | L. IFG (Pars opercularis)  *[-50 24 26], p<0.000*  L. PreMot+ SuppMotor Area  *[-38 6 26], p<0.000*  *L. Hippocampus*  *[-36 -34 -6], p<0.000*  *L. Putamen*  *[-18 8 2], p<0.000* | L. IFG (Pars opercularis)  *[-51 15 24], p<0.000*  L. IFG (Pars triangularis)  *[-54 30 6], p<0.000*  L. dlPFC  [-42 33 12], *p<0.000*  L. PreMot+ SuppMotor Area  *[-54 6 6], p<0.000*  L. Fusiform  [-39 -57 -15], *p<0.000*  *L. Insula*  [-30 21 0], p<0.000 | | | L. IFG (Pars opercularis)  *[-54 24 30], p<0.000*  L. IFG (Pars triangularis)  *[-39 30 3], p<0.000*  L. dlPFC  [-51 30 18], *p<0.000*  L. PreMot+ SuppMotor Area  *[-54 0 48], p<0.000* | L. IFG (Pars opercularis)  *[-42 15 12], p<0.000*  L. IFG (Pars triangularis)  *[-45 24 15], p<0.000*  L. PreMot+ SuppMotor Area  *[-3 12 66], p<0.000* |

1. **Supplementary Results**

**Lateralisation index across time-points**

**Whole cerebral hemisphere (no cerebellum):** LIs decreased over time in both LTLE (from 0.36 pre-surgery to 0.20, 4-months after, to subsequent decrease in the long-term to 0.18), and for RTLE (from 0.55 to 0.39) groups, while controls remained relatively stable over time. These changes were not statistically significant. Controls had statistically higher (more left-lateralised) LIs than LTLE (*F*(3, 60) = 3.13, *p* = .035), and numerically higher LIs than RTLE (not significant).

F**rontal lobe:** LTLE and RTLE showed a decrease in LIs over time, particularly in RTLE (from 0.73 to 0.25). A one-way ANOVA with repeated measures showed that for RTLE there was a significant difference between the LIs across time-point, *F*(3,30) = 8.11, p = .001. Controls had numerically higher (more left) LIs than LTLE (not statistically significant, p= 0.197), and statistically higher LIs than RTLE (*F*(3, 60) = 6.89, *p* = .001).

**Temporal lobe:** Significant decreases in LIs were observed in LTLE (from 0.42 to 0.18; one-way ANOVA with repeated measures: *F*(3,30) = 3.42, p = .033). In RTLE, LIs decreased at the long-term follow up from 0.49 to 0.37. Controls also showed a minor decrease. RTLE and CTRL changes were not significant. Controls had statistically higher (more left) LIs than LTLE (*F*(3, 60) = 5.8, *p* = .002), no differences were found for RTLE.

**Cerebellum:** In LTLE group, cerebellar laterality shifted from right-lateralised to bilateral, then back to right-lateralisation at 12 months post-op and in the long-term. The changes were significant (*F* (3,30) = 8.1, p = .001). RTLE group also showed decrease in the long-term, that was non-significant. Controls remained stable. Controls had more right-lateralised LIs than LTLE, (*F*(3, 60) = 6.2, *p* = .001), no differences for RTLE were significant.
